# The Role of Dietary Fibre in Enteral Nutrition in Sepsis Prevention and Therapy: A Narrative Review

**DOI:** 10.3390/nu15112489

**Published:** 2023-05-26

**Authors:** Valentina V. Huwiler, Melanie Scalise, Katja A. Schönenberger, Stefan Mühlebach, Zeno Stanga, Maria L. Balmer

**Affiliations:** 1 Department of Diabetes, Endocrinology, Nutritional Medicine and Metabolism (UDEM), Inselspital, Bern University Hospital, University of Bern, 3010 Bern, Switzerland; valentina.huwiler@extern.insel.ch (V.V.H.); zeno.stanga@insel.ch (Z.S.); 2 Division of Clinical Pharmacy and Epidemiology, Department of Pharmaceutical Sciences, University of Basel, 4031 Basel, Switzerland; stefan.muehlebach@unibas.ch; 3 Department of Biomedical Research, University Clinic of Diabetes, Endocrinology, Nutritional Medicine and Metabolism, Inselspital, Bern University Hospital, University of Bern, 3010 Bern, Switzerland; 4 Diabetes Center Berne (DCB), 3010 Bern, Switzerland

**Keywords:** enteral nutrition, dietary fibre, sepsis, infection, microbiome

## Abstract

Objective: This narrative review summarises the current evidence on the role of dietary fibre in enteral nutrition in the prevention and therapy of sepsis, with a focus on critically ill patients. The aim is to discuss the implications for clinical practice and identify future directions for policy and research. Resources: We searched MEDLINE and Google Scholar for records on sepsis, critically ill, enteral nutrition, and dietary fibre. We included all types of articles such as meta-analyses, reviews, clinical trials, preclinical studies, and in vitro studies. Data were evaluated for significance and clinical relevance. Synopsis of Review: Despite the ongoing debate, enteral nutrition containing dietary fibres showed great potential in attenuating sepsis-related outcomes and preventing the incidence of sepsis in critically ill patients on enteral nutrition. Dietary fibres target different underlying mechanisms such as microbiota, mucosal barrier integrity, local cellular immune response, and systemic inflammation. We discuss the clinical potential and concerns that currently exist with the standard implementation of dietary fibre in enterally fed intensive care patients. Additionally, we identified research gaps that should be addressed to determine effectiveness and the role of dietary fibres in sepsis itself and its associated outcomes.

## 1. Introduction

Sepsis is a complex and “life-threatening organ dysfunction caused by a dysregulated host response to infection” that can lead to septic shock, multiple organ failure, and death [1]. It is the leading cause of mortality in intensive care units (ICUs), with nearly one in four cases being fatal, and is responsible for nearly 20% of deaths worldwide. In 2017, 48.9 million cases of sepsis were recorded worldwide, making it a significant global health burden. The most common causes of sepsis are infections, accounting for 68.9% of the cases, followed by non-communicable diseases (27.5%) and injuries (3.6%) [2].

Sepsis prevention and treatment strategies differ depending on the stage and course of the occurrence. The primary goal of sepsis prevention is to avoid infections. This can be achieved through strategies such as safe food preparation, improving sanitation and water quality, and adequate nutrition. In case of infections, early detection and prompt treatment (e.g., with antibiotics) are essential. This includes the prevention of microbial translocation through disrupted physiological barriers such as the mucosal membrane of the gastrointestinal tract. In the case of translocation, it is essential to halt the course of sepsis by ensuring an appropriate host response. Signs and symptoms of sepsis may vary depending on the patient and the course of the disease and include fever or low temperature, altered mental status, difficulty breathing, increased heart rate, and weak pulse/low blood pressure [3].

Critically ill patients are prone to sepsis and show complex and fluctuating immune and inflammatory changes. Malnutrition occurs frequently because of increased catabolism in the early period and augmented anabolism in the late period. In such cases, enteral nutrition (EN), which provides nutrients through the gastrointestinal tract, can be an effective way to cover the nutritional requirements of these patients [4]. Dietary fibres (DFs) are a type of indigestible carbohydrate that can be fermented by the gut microbiota to produce short-chain fatty acids (SCFAs), among others, which have pleiotropic beneficial effects on the body [5]. Due to the stimulating effect on protective microbial populations, some DFs can be classified as prebiotics. Prebiotics are often administered together with beneficial bacteria, i.e., probiotics, which are summarised under the term synbiotics [6]. Evidence is accumulating that DFs, as a component of EN, may have protective and therapeutic effects in sepsis. However, their effectiveness, safety, and generalisability remain controversial. This narrative review aims to explore the existing evidence on the role of DFs in EN and its potential to prevent and treat sepsis.

## 2. Methods

This narrative review was based on a literature search of the MEDLINE and Google Scholar databases to identify relevant studies on sepsis, critically ill, EN, and DFs from inception to September 2022. The search terms included the keywords sepsis, critically ill, bacteraemia, fungaemia, viraemia, bloodstream infection, enteral nutrition, tube feeding, dietary fibre, prebiotic, dietary carbohydrate, wheat bran, roughage, resistant starch, inulin, arabinoxylan, xylan, pectin, beta-glucan, guar gum, arabica gum, oligosaccharide, oligofructose, inulin, non-starch polysaccharides, soy polysaccharides, lignin, cellulose, pea fibre, and inulin-type fructans (Appendix A). The types of articles reviewed in this paper covered clinical trials, animal studies, in vitro examinations, reviews, and meta-analyses. We favoured the most clinically accurate literature addressing our research question and up-to-date articles. We included preclinical studies in case no human clinical evidence was available or to underline mechanistic aspects. Studies with a mixed intervention that included a potential confounder in addition to DFs were excluded if a study was found that examined the isolated effect of DFs. Similarly, studies that included other forms of nutrition, such as oral or parenteral nutrition, were excluded if we found a study that examined the effect of enteral nutrition on the same research question. Case studies and clinical trials were excluded if a recent meta-analysis was published on the same research question. Literature for the mode of action was not restricted to search terms to prevent the exclusion of relevant studies.

## 3. Impact of Dietary Fibres on Intestinal Homeostasis

**Figure 1:**
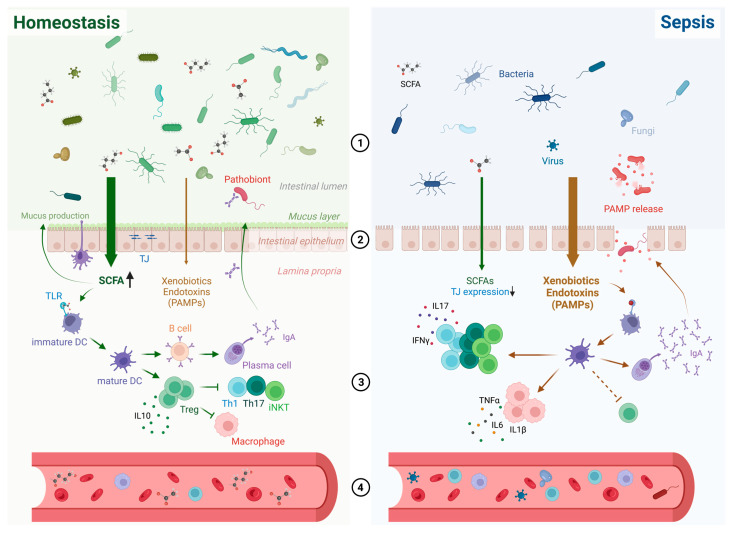
Putative mechanism of dietary fibre in preventing and treating sepsis. Key modes of action are indicated by the numbers 1 to 4. 1. Increase in microbiome diversity and SCFA production, 2. support of intestinal integrity via tight junction expression, 3. improvement of cellular immune responses, 4. regulation of inflammation. DC = dendritic cell, IgA = immunoglobulin A, IL = interleukin, iNKT = invariant natural killer T cell, PAMP = pathogen-associated molecular pattern, SCFA = short-chain fatty acid, Th = T helper cell, TJ = tight junction, TLR = toll-like receptor, TNF-a = tumor necrosis factor alpha, Treg = regulatory T cell. Created with BioRender.com.

A cascade of negative effects including infection, intestinal microbiome dysbiosis, microbial translocation, and dysregulated host immune response may result in sepsis. The role of DFs as part of EN during sepsis could be due to several mechanisms, which are discussed in this section (Figure 1, Table 1). DFs can perform the following functions:

1. Shaping the microbiome composition, diversity, and function [7];
2. Supporting the intestinal barrier integrity [8,9,10];
3. Regulating immune responses in intestinal tissue [10,11];
4. Mediating systemic inflammation [10,11].

**Table 1:** Overview of studies and meta-analyses on dietary fibre supplementation in enteral nutrition and intestinal homeostasis.

Mechanism	Study (First Author, Year)	Study Population and Size	Dietary Fibre Type	Results
**Microbiota**	Freedberg, 2020 [12]	ICU patients with broad-spectrum antibiotics (*n* = 20, I 10/C 10)	Soy- and oat-derived fibre	↑ SCFA stool ↑ SCFA producer
	Majid, 2013 [13]	ICU patients (*n* = 22, I 12/C 10)	FOS/Inulin	= Faecal *Bifidobacterium*
	Simakachorn, 2011 [14]	ICU paediatric patients (*n* = 80, I 41/C 39)	FOS, Inulin, Acacia gum	↑ Faecal *Bifidobacterium* ↑ Total *Lactobacillus* = *Enterobacteriaceae*
**Intestinal barrier integrity**	Liu, 2022 [15]	MA, critically ill patients (ON, EN and PN, *n* = 115, I 63/C 52)	Various	↓ Intestinal permeability
	Spindler-Vesel, 2007 [16]	Trauma patients (*n* = 81, F 29/C 26/S 26)	Guar gum, β-glucan, inulin, pectin, resistant starch (+probiotics)	↓ Intestinal permeability
	Lopez, 2014 [17]	Patients with multi-organ failure (*n* = 89, I 46/C 43)	Dietary fibre (+probiotics)	↓ Mucosa colonisation by *Candida* ↓ Lactate levels
	Wang, 2020 [18]	Mice with sepsis ^1^ (*n* = 48, I 24/C 24)	GOS, stachyose, MOS	↑ Intestinal barrier function
	Aydogan, 2007 [19]	Operated rats (*n* = 24, I 12/C 12)	Cellulose	= BTR = Ileal changes
	Sanchez, 1994 [20]	Rats with enterocolitis (*n* = 72, I 36/C 36)	Pectin	↑ Mucous parameters
	Hou, 2010 [21]	Rats with trauma (*n* = 12, I 6/C 6)	Dietary fibre	↓ Endotoxins in portal vein
**Local cellular immune response**	De Luis, 2002 [22]	Oral and laryngeal cancer patients (*n* = 47, I 23/C 24)	Dietary fibre (+arginine)	= Lymphocytes
	Lee, 2016 [23]	ICU patients (*n* = 15, I 8/C 7)	β-glucan	↑ NK cell activity
	Mao, 2022 [24]	Stroke ICU patients (*n* = 60, I 30/C 30)	Pectin (+probiotics)	= Lymphocytes
	Abe, 2018 [25]	Patients with oesophageal cancer surgery (*n* = 326 (I 137/C 189)	Guar gum, FOS (+glutamine)	↓ L/N ratio
	Hou, 2010 [21]	Rats with trauma (*n* = 12, I 6/C 6)	Dietary fibre	= TNF-α = IL-6 ↑ sIgA
**Systemic** **inflammation**	Liu, 2022 [15]	MA, critically ill patients (ON, EN and PN; *n* = 104, I 53/C 51)	Various	↓ CRP
	De Luis, 2002 [22]	Oral and laryngeal cancer patients (*n* = 47, I 23/C 24)	Dietary fibre (+arginine)	= Albumin = Prealbumin = Transferrin
	Mao, 2022 [24]	Stroke ICU patients (*n* = 60, I 30/C 30)	Pectin	↑ Prealbumin
	Abe, 2018 [25]	Patients with oesophageal cancer surgery (*n* = 326, I 137/C 189)	Guar gum, FOS (+glutamine)	↓ SIRS ↓ CRP
	Olah, 2007 [26]	Patients with severe acute pancreatitis	Dietary fibre (+probiotics)	↓ SIRS

^1^ Oral nutrition of mice, ↑ significant increase, = no significant effect, ↓ significant decrease, BTR = bacterial translocation rates, C = control group, CRP = C-reactive protein, FOS = fructooligosaccharide, GOS = galactooligosaccharide, I = intervention/dietary fibre group, ICU = intensive care unit, IL = interleukin, L/N ratio = lymphocyte/neutrophil ratio, MA = meta-analysis, MOS = mannose oligosaccharide, NK = natural killer, sIgA = secretory immunoglobulin A, SIRS = systemic inflammatory response syndrome, TNF-a = tumor necrosis factor alpha.

### 3.1. Shaping the Composition and Function of the Microbiota through Dietary Fibres (1)

In response to sepsis and critical illness, a sudden and drastic collapse of the commensal microbiota and its replacement by pathobionts has been demonstrated, in line with a decline in microbial diversity [27,28,29] (Figure 1).

#### 3.1.1. Immunological Role of the Microbiota in Health and Disease

The gut microbiota plays an important role in shaping and modulating the host immune system, and commensal bacteria are crucial for protection against infections. The microbial composition and complexity within the gut ensure the ability of the microbiota to generate a consistent immunomodulatory effect within the host and maintain the intestinal gut barrier integrity [30,31]. An alteration of predominant commensal gut microbes and their reduced stability is called dysbiosis and can be associated with the development of several diseases [31,32,33]. It has been shown that microbial composition and complexity vary between healthy and diseased individuals [34,35,36,37,38]. Not only endogenous but also exogenous factors such as host physiology and immunity, diet, and drugs such as antibiotics influence microbial composition [39,40]. Several studies showed that the use of antibiotics early in life leads to alterations of the intestinal microbiota in a murine model and that these alterations recovered after cessation of antibiotics, while the altered metabolic phenotype caused by the application of antibiotics persisted during adulthood [41,42,43,44]. Others showed how the use of antibiotics strongly affected the gut microbial composition and promoted a pro-inflammatory phenotype in the long term by changing the frequency and function of invariant natural killer T cells even after reconstitution of the gut microbiome [42,45,46].

#### 3.1.2. Beneficial Effect of Dietary Fibre Fermentation through the Gut Microbiota

Diet is an important exogenous factor reshaping the intestinal microbiota [47]. A westernised diet has been characterised as consisting of a high fat content but a low DF content [10,31]. There are several studies implementing the effect of diet, especially a low-DF westernised diet, on gut microbial composition contributing to an altered host immune response in murine models and in humans [10,31,48,49,50]. During DF fermentation by commensal gut bacteria, SCFAs such as acetate, propionate, and butyrate are produced [51,52]. Dietary changes such as high-DF diets have been associated with an increase in SCFA production within the gut [53]. SCFAs have been shown to have a beneficial effect on the intestinal gut barrier integrity and regulatory T cell (Treg) differentiation which will be discussed later in more detail.

#### 3.1.3. Impact of Dietary Fibre Supplementation on the Intestinal Microbiota during Enteral Nutrition in Sepsis

A preclinical study showed that the diversity of the faecal microbiota in mice with sepsis increased after oral DF supplementation compared to the standard diet [18]. Two clinical studies in enterally fed ICU patients showed an increase in SCFA-producing bacteria, *Bifidobacterium,* and *Lactobacillus*, as well as increased levels of SCFAs in the stool, with no effect on *Enterobacteriaceae* populations upon DF supplementation [12,14]. Another study with a similar DF found no significant effect on faecal *Bifidobacterium* abundance [13] (Table 1).

### 3.2. Supporting the Intestinal Barrier Integrity through Dietary Fibres (2)

Maintaining the intestinal gut barrier integrity is fundamental to prevent the translocation of microorganisms and endotoxins to the systemic circulation, which could eventually lead to sepsis [54] (Figure 1).

#### 3.2.1. Structure and Function of Intestinal Barrier

The intestinal barrier consists of a single layer of cells, forming an interface between luminal intestinal microorganisms and the host immune system, and represents the first line of defence against intestinal microorganisms and other environmental factors [10,45,55]. The intestinal epithelium earns its barrier function through tight junctions (TJs) which are intercellular adhesion molecules controlling paracellular permeability [56]. A second key player contributing to the intestinal barrier function is the mucus layer produced by so-called goblet cells (GCs) which are located within the intestinal epithelium [55]. Several studies showed that mice lacking the *Mucin 2 gene* (*MUC2*), a glycoprotein produced by GCs that strengthens the gut barrier integrity, were more susceptible to colitis since pathobionts could easily translocate through the intestinal barrier [57,58,59].

#### 3.2.2. Beneficial Effect of DF Fermentation on the Intestinal Barrier Integrity

It has been previously shown that SCFAs are able to suppress intestinal inflammation in a murine model of colon cancer [39,49] and promote mucus secretion [48,60]. Acetate, an SCFA deriving from *Bifidobacteria* among others, has been shown to influence GC differentiation in gnotobiotic rats [61]. Arike and colleagues were able to show that germ-free mice lacking SCFAs show shorter *MUC2* O-glycans, which correlated with the decrease in the respective glycosyltransferase enzymes responsible for glycan elongation [62]. One example of the beneficial effects of SCFAs has been shown by microbial-derived butyrate, which enhances intestinal barrier integrity in mice [39,63]. Furthermore, SCFAs promoted the secretion of immunoglobulin A (IgA) by B cells [10,48,64]. IgA plays a key role in maintaining gut homeostasis by regulating the host immune system towards tolerance of the commensal gut microbiota rather than responsiveness [10,45,48]. Liu and colleagues also showed that oral administration of SCFAs promoted gut homeostasis in shifting the T cell response towards an anti-inflammatory phenotype by promoting the secretion of interleukin 10 (IL-10) by Treg cells [33]. In addition, several studies showed that environmental factors can affect intestinal homeostasis [10,31,65,66,67]. For example, the use of antibiotics resulted in impaired intestinal barrier integrity [46,68], while a high-fat or westernised diet has been associated with gut microbial dysbiosis. This results in a decreased abundance of SCFA-producing bacteria [37,69] or bacteria promoting mucus secretion [48,61] and an increased intestinal barrier permeability by decreasing TJ expression [70]. Therefore, a leakage of toxic bacterial components such as LPS into the bloodstream could be observed, causing endotoxinaemia [10,31,43,71,72,73]. Simpson and colleagues demonstrated that a westernised diet low in DFs increases the abundance of mucosa-penetrating *Proteobacteria* [7,10]. Further, it has been shown that a westernised diet alters the gut microbial diversity, resulting in a loss of *Bacteriodetes* [7,10,74]. Several studies identified beneficial effects of *Akkermansia muciniphila*, *Bifidobacterium* spp., *Bacteroidetes* spp., *Lactobacillus* spp., and *Clostridiales* spp. as gut barrier-promoting bacteria, while *Oscillibacter* spp. and *Desulfovibrio* deteriorated the integrity of the intestinal barrier [31,71,75,76,77,78,79,80]. In addition, Ding and colleagues found that transplanting gut microbiota from mice fed a high-fat diet to germ-free (GF) mice activated the pro-inflammatory pathway (NFkb1), indicating that diet-induced dysbiosis is sufficient to cause intestinal inflammation [31,81]. Dietary interventions such as supplementation of DFs have been shown to promote gut barrier integrity in mice through an increased SCFA production [52,53].

In addition, DFs promote gut homeostasis and intestinal barrier integrity independent of the microbiota by interacting with intestinal epithelial cells and immune cells. Depending on the type of DF, epithelial TJ protein, GC function, or epithelial cell and glycocalyx maturation can be modulated. While there is high evidence for HMOs and galactooligosaccharides, no such effect has been observed for arabinoxylan and β-glucan [82].

#### 3.2.3. Impact of Dietary Fibres on the Intestinal Barrier Integrity during Enteral Nutrition in Sepsis

Intestinal permeability was significantly reduced in a preclinical study in mice with sepsis by oral feeding containing DFs [18]. Two studies in enterally fed rats with trauma and enterocolitis indicated similar improvement in intestinal barrier function [20,21]. However, the supplementation of cellulose to EN could not improve barrier function in operated rats [19]. In two clinical studies by Spindler-Vesel (2007) and Lopez (2014), intestinal permeability decreased after symbiotic treatment and prebiotic treatment, respectively, compared to control diet [16,17]. This reduction in intestinal permeability was confirmed in a meta-analysis by Liu et al. in critically ill patients without restrictions on the route of nutrition [15] (Table 1).

### 3.3. Regulation of the Local Cellular Immune Response by Dietary Fibres (3)

A dysregulated host immune response to infection is a key factor of sepsis according to the third international consensus definitions [1]. DFs are metabolised by beneficial gut bacteria, such as *Bifidobacteria* and certain *Bacteroides* species, resulting in a release of microbial metabolites. These microbial metabolites such as butyrate or acetate can serve as an energy source for colonic epithelial cells and modulate metabolism and immune function [51,83] (Figure 1).

#### 3.3.1. Mechanism of Local Cellular Immune Response Related to Dietary Fibres

Under normal conditions and if gut intestinal barrier integrity persists, the immune system is able to balance between elimination of pathobionts and tolerance towards beneficial commensal gut bacteria [47,55,59]. Even commensal gut bacteria need continuous monitoring by the intestinal immune system to prevent their outgrowth and mischief [84,85,86,87]. Antibody-presenting cells (APCs), such as dendritic cells (DCs), play an important role in monitoring the gut microbiota. According to which pathogen-associated molecular patterns (PAMPs) they sense, DCs can distinguish commensal microbes from pathobionts and activate the corresponding downstream immune responses [84]. During homeostasis, DC maturation occurs upon SCFA binding among others, resulting in antigen presentation to naïve T cells and differentiation into Treg cells [33]. Several studies emphasise the influence of butyrate on gene expression by the inhibition of histone deacetylases which influences the differentiation of naïve T cells into Treg cells in mice [55,63,65,88,89,90]. Treg cells are important regulatory cells for dampening a pro-inflammatory immune response and therefore avoiding excessive inflammation by secreting anti-inflammatory IL-10 responsible for an immunosuppressive effect [33,91].

Older literature already showed evidence on IgA production, which is highly dependent on the presence of intestinal gut microbes since mucosal IgA levels in GF mice were very low but could be restored after colonisation of these GF animals [92,93,94]. This indicates that IgA may play an important role in maintaining the symbiotic relationship between the host and the gut microbiota [48,95,96,97]. Upon activation by PAMPs or SCFAs via DCs, B cells enter the systemic circulation through the lymph and the blood to then home back to the intestinal mucosal tissue and seed it with IgA-secreting plasma cells [95]. Once the intestinal lumen is reached, IgA prevents bacterial invasion by binding to specific microbes (commensal bacteria to avoid outgrowth or pathobionts to avoid infections) and therefore blocking the attachment to the host [10,94,95,98]. This binding of IgA to microbes marks bacteria for phagocytosis and antigen presentation to DCs [10]. Several studies show indirect evidence of SCFAs promoting the secretion of IgA by B cells [48,64].

DCs can undergo maturation and activation also through other factors, such as diet or PAMPs derived from pathobionts. After DC activation and maturation, antigens are presented to naïve T cells, resulting in their proliferation and differentiation into pro-inflammatory effector T cells, such as Th1, Th17, and invariant natural killer T cells, rather than Treg cells [47].

The communication between the intestinal microbiota and the immune system plays a crucial role in gut homeostasis, and even the smallest changes in their communication may lead to the onset of a disease, microbial dysbiosis, alterations in bacteria-derived metabolites, and impaired intestinal barrier integrity, which then lead to activation of macrophages and DCs, giving rise to a pro-inflammatory immune milieu. Reduced microbial tolerance has been associated with many diseases [47,70,99,100,101,102,103,104]. Park and colleagues showed that if the host was in a situation of fighting against pathobionts, the bacteria-derived SCFAs shifted the immune system towards pro-inflammatory Th1/Th17 effector T cells in order to boost immunity [91,105].

#### 3.3.2. Impact of Dietary Fibres on the Local Immune Response during Enteral Nutrition in Sepsis

Preclinical studies in rats with trauma showed an increase in secretory IgA but no effect on TNF-α and IL-6 in EN with DFs compared to standard EN [21]. There were also conflicting results in two clinical studies where natural killer cell activity was increased [23,24] but there were no significant effects on lymphocytes [22,24] (Table 1).

### 3.4. Mediation of Systemic Inflammation by Dietary Fibres (4)

During sepsis, DFs might modulate pro- and anti-inflammatory parameters and therefore prevent the development of systemic inflammation (Figure 1).

#### 3.4.1. Development of Local to Systemic Inflammation

It has been shown that at sites of infections, the levels of SCFAs, such as acetate, increased and modulated the subsequent immune responses by increasing IL-10, decreasing pro-inflammatory interferon gamma (IFNγ) and tumor necrosis factor alpha (TNFα) [106], and being distributed systemically [48,107,108]. These findings indicate the importance of metabolites such as SCFAs or bile acids (BAs) on the systemic immune response. Indeed, several studies indicate that the effect of SCFAs is not only limited to the gut [105], which will be discussed later in more detail.

BAs derive from cholesterol in the liver and are secreted into the duodenum and further modified by the gut microbiota in the intestine, resulting in so-called secondary BAs, where they act as potent signalling molecules [35,101]. One main task of BAs is the emulsification of lipids, which explains why BA secretion is enhanced upon a high-fat diet in order to facilitate lipid digestion [109,110]. Another important feature of BAs is their antimicrobial activity [109,111]. Therefore, BAs are able to shape the gut microbiome to their favour, e.g., by promoting BA-metabolising bacteria and preventing the growth of bacteria sensitive to BAs [31,109,112]. BAs have several metabolic effects through their interaction with the farnesoid X receptor (FXR) and TGR5 [53]. Through activation of FXR and TGR5, BAs are able to promote glycogen synthesis and insulin sensitivity in the liver; further, they increase insulin secretion by the pancreas and promote satiety in the brain [53,113,114].

#### 3.4.2. Role of Dietary Fibres in the Mediation of Systemic Inflammation

High-DF diets have been shown to protect against experimental intestinal inflammation and interact with the host immune responses [45,115,116].

As already discussed earlier, microbial-derived metabolites such as SCFAs influence many distant organs and their immune responses, while diet plays an important role in shaping the gut microbiome, including SCFA-producing microbes. Thornburn and colleagues showed that a high-DF diet resulted in high amounts of acetate and suppressed allergic airway disease by enhancing Treg cell immune responses [105,117]. Further, high-DF diets and subsequent production of propionate have been shown to induce haematopoiesis of DCs and reduce a Th2 immune response [105,118]. In the meantime, Braniste and colleagues showed the effect of SCFAs on the blood–brain barrier (BBB), where a colonisation with butyrate-producing *Clostridium tyrobutyricum* or acetate and propionate-producing *Bacteroides thetaiotaomicron* decreased BBB permeability [119].

Regarding the impact of DFs on BAs and the following downstream mechanisms, the literature presents evidence on the different types of DFs. Bretin and colleagues identified psyllium, a semi-soluble DF protecting against colitis via altering BA metabolism through FXR activation, which suppresses pro-inflammatory signalling pathways [120]. Others showed that different soluble DFs such as inulin and pectin were able to protect against diet-induced obesity but exacerbate experimental colitis [120,121,122]. Other papers support the fact that the effect of DFs highly depends on the type of DF by showing that inulin DF diets can trigger eosinophilia and Th2 immune cell response not only in the intestine but also in the lungs, which in excess are hallmarks for allergic asthma [123]. Further, Zhou and colleagues showed that some insoluble DFs such as bamboo shoot DF increased SCFA and BA levels influencing lipid metabolism in mice fed a high-fat diet [124]. Furthermore, a low-DF westernised diet was associated with BA profile dysregulations contributing to the development of chronic inflammatory diseases such as diabetes type 2 and colon cancer, and this might be alleviated by DF supplementation since soluble DFs increase the SCFA levels [50,125,126].

#### 3.4.3. Impact of Dietary Fibres on the Systemic Inflammation during Enteral Nutrition in Sepsis

EN with DFs consistently reduced systemic inflammatory response syndrome (SIRS) and C-reactive protein (CRP) levels in critically ill patients compared to standard EN [17,25,26]. The meta-analysis by Liu et al. (2022) confirmed the reduction in CRP levels by DF supplementation in critically ill patients [15]. Albumin, prealbumin, and transferrin levels remained unchanged [22], and prealbumin levels even increased in stroke ICU patients after treatment with pectin-containing EN [24] (Table 1). 

## 4. Clinical Benefits of Dietary Fibre Supplementation in Sepsis

Sepsis is not only a life-threatening condition itself but is closely interlinked with several adverse clinical outcomes, such as diarrhoea, infection, extended length of hospital and ICU stay, and mortality [2]. Based on the potential beneficial effects of DFs on gut homeostasis, we discuss in the following section how DF supplementation may improve sepsis outcomes in clinical care (Table 2).

### 4.1. Dietary Fibre and Sepsis

Critically ill patients often show complex and fluctuating immune and metabolic states. Malnutrition occurs frequently because of increased catabolism in the early period and augmented anabolism in the late period. Oral nutrition is rarely sufficient in these patients, and administration of nutrients via the enteral or even parenteral routes is needed to cover their nutritional requirements [4]. Adding DFs to EN not only improves the above-mentioned underlying causes of sepsis but could also prevent the development of sepsis. There is no meta-analysis addressing exactly this question; however, the meta-analysis by Chi et al. (2019) showed a decrease in the relative risk for sepsis in pre-term infants when prebiotics were supplemented orally, enterally, or parenterally [127]. Li et al. (2021) found that prebiotics together with probiotics significantly decreased the incidence of sepsis in critically ill adults, whereas the effect of prebiotics alone remained insignificant [128]. A single clinical trial could show a significant decrease in catheter-related sepsis when EN with DFs was used compared to standard EN [129] (Table 2).

### 4.2. Dietary Fibre on Diarrhoea

Diarrhoea is commonly defined as the passing of at least three unformed stools or more than 250 g of unformed stool per day, often in addition to other bowel symptoms [137]. It is a frequent symptom of many infections that can cause sepsis but also a symptom of sepsis itself. A dysregulated host immune response can trigger inflammation and mucosal necrosis and lead to diarrhoea [138]. Among all age groups, sexes, and locations, diarrhoeal disease is the most common underlying cause of sepsis [2]. Managing diarrhoea while avoiding constipation in ICU patients remains a major challenge where DFs could be an important step towards success. Several clinical studies have shown that EN containing DFs decreased diarrhoea and reduced the number of liquid stools compared to standard EN. Four meta-analyses summarised the current evidence, whereof three concluded that EN with DFs decreased the incidence of diarrhoea [128,130,132] and one showed no significant effect [131] (Table 2).

### 4.3. Dietary Fibre and Infection

Infections are the precursors of sepsis where organ dysfunction and dysregulated host responses are not yet present [139]. Controlling infections at an early stage has great potential to reduce the incidence of sepsis. In a study on 172 patients with major abdominal surgery, EN containing DFs and probiotics significantly reduced rates of bacterial infections compared to standard EN formulas [133]. Similarly, a meta-analysis showed a significant reduction in infections in pre-term infants after synbiotic treatment, whereas DFs alone did not show any significant effect [140] (Table 2).

### 4.4. Dietary Fibre and Length of Hospital or ICU Stay

Each hospitalisation for sepsis is estimated to cost USD 35,000, contributing greatly to the rising healthcare costs worldwide [141]. Reducing the length of hospital or ICU stay could decrease these costs immensely. Two studies, one on very low birthweight neonates and one on ICU patients, found significantly reduced length of hospital and/or ICU stay with EN containing DFs compared to standard EN [134,135]. A study on ICU patients with EN and synbiotics could not detect a significant reduction in the length of ICU stay [136] (Table 2).

### 4.5. Dietary Fibre and Mortality

In 2017, 11 million sepsis-related deaths were recorded worldwide, making sepsis a major cause of global death [2]. The ICU mortality and in-hospital mortality decreased significantly for ICU patients that received EN with DFs together with arginine compared to standard EN [129]. Similarly, the mortality was decreased for very low birthweight neonates and pre-term infants who received DFs [127,134] (Table 2).

## 5. Implication for Clinical Practice and Future Research

### 5.1. Clinical Potential

EN compared to parenteral nutrition already prevents and improves sepsis-related outcomes [142,143]. Improvement in EN composition could enhance intestinal barrier integrity, reduce bacterial translocation, and ultimately prevent the occurrence of sepsis. In recent years, clinical evidence that DFs can improve and prevent underlying causes of sepsis has increased drastically. The composition and diversity of the microbiome can be enhanced, resulting in increased levels of SCFAs. Tanes and colleagues showed that a lack of DFs in exclusive EN formulas leads to the slower recovery of the gut microbiome after environmental stress [144]. Although these findings were obtained in healthy individuals, they may be transferable to critically ill patients who are also exposed to high levels of stress and antibiotics. EN with DFs could help to shift the gut microbial composition from dysbiosis back towards eubiosis and improve recovery from sepsis. In addition, most studies have shown that EN with DFs strengthens the intestinal barrier function and decreases permeability. This implies a high potential to inhibit microbial translocation.

Dysregulation of host responses could be reduced, especially in combination with other immunomodulatory nutrients. Interestingly, the meta-analysis by Li and colleagues indicates that sole prebiotic or probiotic supplementation during EN failed to significantly decrease the odds ratio for sepsis. However, when administered together as a synbiotic, the odds ratio was significantly lower compared to patients on standard EN [128]. Many studies have investigated the effect of DFs in EN in addition to other immunonutrients, such as arginine and probiotics, which may overestimate the effect of DFs.

With regard to the consequences of sepsis, DFs have a high potential to reduce diarrhoea in enterally fed critically ill patients. Since diarrhoea is the most common cause of sepsis, this potential should not be overlooked. Clinical evidence suggested that natural diets reduce the incidence of diarrhoea compared with commercial enteral diets and should therefore be considered in addition [145,146]. Data on efficacy in terms of infections, mortality, and shortening of length of hospital or ICU stay are limited, especially regarding the effect of DFs alone during EN. The present results are promising and show a positive trend, but further research is urgently needed. The same applies to the prevention of sepsis.

### 5.2. Concerns Regarding Dietary Fibre Supplementation

The European Society for Clinical Nutrition and Metabolism (ESPEN) guidelines lack recommendations on the use of DFs for critically ill patients [4]. The American Society for Parenteral and Enteral Nutrition (ASPEN) and the Society of Critical Care Medicine (SCCM) recommend fermentable soluble DF supplements to be considered in stable medical and surgical ICU patients but advise against the routine use of mixed or insoluble DFs [147]. There are particular concerns in patients at high risk of bowel ischaemia or severe dysmotility due to reported bowel obstruction in surgical and post-traumatic patients receiving EN containing insoluble DFs [148,149]. These haemodynamically unstable patients may be susceptible to feeding intolerances. Increased fermentation and bowel distention can occur, which results in an increased risk of adverse events [147]. DFs as a main source of fermentation could enhance negative effects. However, clinical evidence undermining these concerns is scarce.

DFs may be of limited benefit in patients receiving oral antibiotics, and close monitoring of potential side effects, such as abdominal pain or flatulence, is indicated. The faecal microbiota of healthy volunteers exhibited decreases in diversity, richness, and evenness after a 5-day administration of oral ciprofloxacin [150]. Another study with a 7-day clindamycin administration showed that the *Bacteroides* population, known for its high DF-degrading ability, does not return to its original composition for up to 2 years after treatment. In phases of a reduced abundance of DF-degrading microbes, the DFs may not be able to unfold their full beneficial effects.

Another important point to consider is the interaction between DFs and drugs, which is discussed elsewhere [151,152].

## 6. Conclusions and Future Directions

There is growing evidence that the benefits of EN containing DFs outweigh the risk in most patients, as they target different underlying mechanisms such as mucosal barrier function, cellular defence, and inflammation.

However, the clinical evidence on the effect of EN containing DFs on sepsis and the associated outcomes is scarce and insufficient. Further research with large and high-quality clinical trials on the effect of DFs alone without other immunonutrients is needed to answer these questions. The type of DF (soluble or insoluble) may also play a role in the beneficial effect of DFs and should be considered. In addition, the role of non-fermentable, insoluble DFs (e.g., cellulose, lignin) should be investigated in more detail, along with the potential additive effect of natural enteral-feeding rich in DFs.

Beyond that, there is a high potential for the supplementation of bacterial metabolites such as butyrate or acetate to bridge the phases of a deprived microbiota. Clinical results on these postbiotics remain scarce.

## Figures and Tables

**Table 2:** Overview of studies and meta-analyses on dietary fibre supplementation in enteral nutrition and clinical benefits.

Outcome	Study	Study Type	Study Population	Population Size	Background Treatment	Results for MA: RR/OR/MD (95% CI)
**Sepsis**	Chi, 2019 [127]	MA	Pre-term infants (ON, EN, PN)	1106	-	↓ RR 0.64 (0.51, 0.78)
	Li, 2021 [128]	MA	Critically ill adults (ON, EN, PN)	525	Probiotics	= OR 0.55 (0.15, 1.90) ↓ OR 0.34 (0.16, 0.70)
	Caparros, 2001 [129]	RCT	Critically ill patients	220	-	↓ Catheter-related sepsis RR 0.07 (0.01 to 0.54)
	Cara, 2021 [130]	MA	Hospitalised critical care patients	186	-	↓ MD −2.78 (−4.10, −1.47)
	Li, 2021 [128]	MA	Critically ill adults (ON, EN, PN)	7199	Probiotics	↓ OR 0.24 (0.05, 0.94)
	Kamarul Zaman, 2015 [131]	MA	Critically ill adults	936	-	=OR 0.89 (0.41, 1.92)
	Del Olmo, 2004 [132]	MA	Critically ill and post-surgery patients	NA	-	↓ OR 0.66 (0.46, 0.95)
**Infections**	Li, 2021 [128]	MA	Critically ill adults (ON, EN, PN)	4357	Probiotics	= RR 0.65 (0.35, 1.15) ↓ RR 0.37 (0.22, 0.61)
	Rayes, 2002 [133]	RCT	Major abdominal surgery patients	172	Probiotics	↓ Bacterial infections
**Length of Hospital/ICU stay**	Dilli, 2015 [134]	RCT	VLBW neonates	200	-	↓ Length of ICU stay
	Xi, 2017 [135]	RCT	ICU patients	125	-	↓ Length of ICU/hospital stay
	Dehghani, 2022 [136]	RCT	ICU patients	92	Probiotics	=Length of ICU stay
**Mortality**	Chi, 2019 [127]	MA	Pre-term infants (ON, EN, PN)	924	-	↓ RR 0.58 (0.36, 0.94)
	Dilli, 2015 [134]	RCT	VLBW neonates	200	-	↓ Mortality
	Caparros, 2001 [129]	RCT	Critically ill patients	220	Arginine	↓ ICU-mortality ↓ In-hospital mortality

↑ significant increase, = no significant effect, ↓ significant decrease, EN = enteral nutrition, MA = meta-analysis, MD = mean difference, ON = oral nutrition, OR = odds ratio, PN = parenteral nutrition, RCT = randomised controlled trial, RR = relative risk. Meta-analyses were preferred over RCTs.

## Appendix A

Search EN & Fibre & Critically Ill
Search Term MEDLINE
Ovid MEDLINE(R) ALL <1946 to 8 September 2022>

1. Enteral Nutrition/ → 21,494
2. (((Enteral or tube* or force or gastric) adj3 (nutrition or feed*))).ti,ab,kf. → 27,042
3. Exp Dietary fiber/or dietary carbohydrates/or prebiotics/ → → 47,334
4. ((diet* adj3 fib*) or (wheat adj3 bran*) or roughage* or prebiotic* or (dietary adj3 carbohydrate*) or (resistant adj3 starch) or “alimentary fib*” or “stimulance multi fib*” or alant* or “dahlin” or inulin* or “synanthrin” or xylan* or arabinoxylan or xyloarabinan or hemixylan or (beta adj3 glucan*) or “beta dextroglucan” or macrogard or Pectin* or Methoxy?pectin or guar or Glucotard or slocose or supercol or “cyamopsis gum” or decorpa or fibraguar or galactasol or “gum cyamopsis” or “hepart hp 7000” or lejguar or prefill or galacto?oligosaccharide* or “galactose oligomer” or oligogalactose or GOS or fructo?oligosaccharide* or Idolax or “Raftilose P95” or neosugar or oligofructose or Metamucil or Plantaglucide or Ispaghul* or (Plantago adj Seed*) or Iso?gel or Reguval or agiocur or arcolax or betajel or fybogel or konsyl or metamucil or mucilax or mucilose or mucofalk or “plantaginis semen” or plantaglucid* or “plantago ovata extract” or “plantago ovata seed” or psyllium or regulan or transilane or “vi siblin” or volcolon or Flax* or Linum* or Lin?seed*).ti,ab,kf. → 93,681
5. Sepsis/or Critical Illness/ → 102,223
6. (Immuno* or sepsis or septic* or (bloodstream adj infection*) or pyemia* or pyaemia* or (blood adj3 poisoning*) or bacteremi* or fungemi* or parasite* or viremi* or (critical* adj ill*) or (critical* adj car*) or ICU or (intensiv* adj care*)).ti,ab,kf. → 2,636,058
7. 1 or 2 → 35,898
8. 3 or 4 → 123,642
9. 5 or 6 → 2,654,657
10. 7 and 8 and 9 → 205

Search Term Google Scholar
“Enteral nutrition” “Dietary fiber”|Prebiotics “Critical Illness”|Sepsis|Immunonutrition
